# Geographical Inequalities in Mortality by Age and Gender in Italy, 2002–2019: Insights from a Spatial Extension of the Lee-Carter Model

**DOI:** 10.1007/s40980-026-00161-x

**Published:** 2026-05-01

**Authors:** Francesca Fiori, Andrea Riebler, Sara Martino

**Affiliations:** 1 Department of Social Work and Social Policy, University of Strathclyde, Glasgow, United Kingdom; 2 Department of Mathematical Sciences, Norwegian University of Science and Technology, Trondheim, Norway

**Keywords:** Mortality, Spatial inequalities, Lee-Carter model, Approximate Bayesian inference, Italy

## Abstract

Italy reports some of the lowest levels of mortality in the developed world. Recent evidence, however, suggests that even in low-mortality countries improvements may be slowing and regional inequalities widening. This study contributes new empirical evidence to the debate by analysing mortality data by single year of age for males and females across 107 provinces in Italy from 2002 to 2019. We extend the widely used Lee–Carter model to include spatially varying age-specific effects, and further specify it to capture space–age–time interactions. The model is estimated in a Bayesian framework using the inlabru package, which builds on INLA (Integrated Nested Laplace Approximation) for non-linear models and facilitates the use of smoothing priors. This approach borrows strength across provinces and years, mitigating random fluctuations in small-area death counts. Results demonstrate the value of such a granular approach, highlighting the existence of an uneven geography of mortality despite overall national improvements. Mortality disadvantage is concentrated in parts of the Centre–South and North–West, while the Centre–North and North–East fare relatively better. These geographical differences have widened since 2010, with clear age- and gender-specific patterns, being more pronounced at younger adult ages for men and at older adult ages for women. Future work may involve refining the analysis to mortality by cause of death or socioeconomic status, informing more targeted public health policies to address mortality disparities across Italy’s provinces.

## Introduction

Over the past century, high-income countries have seen remarkable improvements in survival, though these gains have been uneven across social group and places (Vallin and Meslé, 2004; Mackenbach, 2012; Mackenbach et al., 2012). More recently, however, mortality improvements have slowed or stalled in many countries, and in some cases have even reversed (Dowd et al., 2024; Kabir & O’Brien, 2023). A growing body of research suggests that this slowdown has been accompanied by increasing geographical dispersion in life expectancy within countries, even among those with historically low mortality levels (Hrzic et al., 2020; Sauerberg et al., 2024). Italy provides a particularly informative case in this context, as high national longevity coexists with substantial and persistent sub-national inequalities, raising the question of where within the country progress has slowed, and for whom.

According to the most recent estimates, the country indeed reports some of the lowest mortality levels in Europe, with a life expectancy at birth of 83.4 years in 2023–surpassing the EU27 average of 81.4 (EUROSTAT, 2025). This remarkable overall achievement, however, conceals the existence of geographical inequalities: for example, there are nearly four years differences in life expectancy at birth between provinces in the South (e.g., 80.9 in Caserta – Campania) and in the Centre-North (e.g., 84.7 in Firenze – Toscana) (ISTAT, 2024). These differences in mortality likely reflect the existence of well-documented geographical disparities in wealth, educational levels and employment opportunities (Daniele & Malamina, 2011); in the concentration of economic activities such as heavy manufacturing industry, with associated environmental risks (De Dominicis et al., 2013); in the availability and quality of healthcare services associated with a decentralised health system (Balia et al., 2018; Benassi et al., 2025).

Moreover, despite its comparatively low mortality levels, the country has experienced some slowdown in the pace of mortality decline since 2010. This deceleration has been linked to the diminishing rate of progress in reducing cardiovascular disease-related deaths (OECD, 2020), the “double jeopardy” (Abrams et al., 2023) of slower improvements at both younger (25–40) and older (55–75) ages (Dowd et al., 2024), and smaller-than-expected gains in longevity for women (Djeundje et al., 2022). Changes in mortality patterns by age, gender and underlying cause, may also find expression geographically, with some areas and population groups experiencing slower progress than others.

This study intends to make an empirical and methodological contribution to understandings of inequalities in contemporary mortality trends in high-income countries. First, it uses mortality data for 107 Italian provinces to study geographical variations in mortality trends beyond population aggregates, studying age-specific mortality rates by gender. Second, it demonstrates the opportunities offered by Bayesian spatial approaches (Goes, 2024) and by recent computational advancements (Rue et al., 2009) to overcome the challenges of producing estimates at a fine geographical detail broken down by one or more characteristics (such as age, gender, time). Specifically, it estimates a spatial extension of the traditional Lee-Carter model (Basellini et al., 2023; Lee & Carter, 1992) to address the following research questions:

1. How has mortality by age and gender evolved over the past two decades?
2. Are there geographical inequalities in mortality rates by age and gender?
3. And have geographical inequalities widened during the slowdown of survival improvement of the 2010s?

Our spatially extended Bayesian Lee–Carter model moves beyond descriptive summaries by stabilizing noisy provincial estimates through spatial smoothing, quantifying uncertainty, and modeling spatio-temporal dependencies, allowing robust and generalizable insights into sub-national mortality dynamics by age and gender.

## The Changing Geography of Mortality in Italy

Demographic research has extensively documented geographical differences in mortality across Italy during the second half of the 20th century [e.g.,Capocaccia and Caselli (1972); Caselli and Egidi (1979, 1981); Caselli (1983); Caselli and Reale (1999); Caselli et al. (2003)]. While highlighting general improvements in survival, this body of research reveals a distinct gendered geography of mortality. Among men, high mortality was initially concentrated in the North–largely due to environmental risks associated with rapid industrialization–but later spread to parts of the Centre-South, particularly Campania. In contrast, the South and the Islands remained the regions with the greatest advantage. For women, mortality, especially among the elderly, was highest in the Apennine region, Sicily, and parts of the North, while the Centre-North was the least affected. Since the 1970s, the decline in mortality has reshaped these patterns. Although the North–South divide persisted for men, high-mortality zones in the North contracted, while Campania and Sicily became new areas of concern. For women, mortality patterns diverged from those of men, with the highest rates now concentrated in Campania and Sicily, particularly among the elderly (Caselli et al., 2003). Cause-specific mortality trends further qualify these gendered patterns. In the wealthier North, men exhibited higher rates of cancer and ischemic diseases, whereas in the less affluent South, mortality was elevated for conditions that could be mitigated through better healthcare. Among women, the South recorded the highest mortality from circulatory diseases and diabetes, while cancers and ischemic heart diseases played a lesser role [e.g., Caselli and Egidi (1981); Caselli et al. (2003)].

Collectively these studies demonstrate that geographical analyses can yield valuable insights, particularly for informing public policy. When combined with analyses of temporal changes, and focused on specific diseases or population age groups, they suggest plausible influence of underlying social, cultural, and environmental factors in different areas. Drawing on observed trends, some of these studies (Caselli et al., 2003) concluded by suggesting that the following century would have ushered a new geographical landscape, particularly for adult and elderly male mortality, reinforcing the need for continued research into spatial variation.

It is therefore no surprise that geographical variations in mortality across Italy have continued to attract scholarly attention in the early twenty-first century. A growing body of research has revisited and updated earlier findings. Notable contributions advancing the debate include recent work by Carboni et al. (2024) and Martin et al. (2025), which analysed spatial convergence in life expectancy using data up to 2019. Their studies suggest a reversal of previous convergence trends, with mortality disparities widening in recent years, particularly at younger adult ages. Although the Northern and Central regions–particularly the North East–have experienced greater improvements in life expectancy, the South and the Islands have lagged behind. This shift has been attributed to healthcare decentralization and financial constraints, especially following the 2008 economic crisis, which disproportionately affected poorer Southern regions (Carboni et al., 2024; Salinari et al., 2023). Similar findings emerge from Sauerberg et al. (2024), who examined provincial data from 16 European countries, including Italy. Their study confirms that despite overall improvements, sub-regional disparities have increased in recent years. Notably, mortality rates for men in previously disadvantaged areas of Northern Italy have aligned with national averages, while Southern regions continue to experience higher mortality rates for both genders (Culotta, 2021). Similarly, Santos Sánchez et al. (2020), using municipal data from 2012 to 2016, identified mortality hotspots in Southern Italy linked to high unemployment levels. Baldi et al. (2026) further qualified these trends by highlighting different trajectories in the so-called deaths of despair. While Northern regions experienced greater declines in alcohol-related mortality, the South showed slower progress, particularly following the economic crisis.

While these studies provide important insights on features and changes of the recent geography of mortality in Italy, they are limited in the extent to which they isolate the contribution of specific aspects to overall patterns. A recent reflection paper on the present and future of mortality studies (Dowd et al., 2024) emphasized the important contribution of age- and cause-specific mortality to understand current and future trends, and the extent to which they reflect biological limits or social, environmental and structural factors that are amenable to change.

Indeed, mortality by leading cause of death can offer a more accurate and nuanced measure of regional differences in economic development and social wellbeing. Using mortality data around 2011, Barbi et al. (2018) and Lagona et al. (2020) demonstrate that the geography of mortality from cardiovascular diseases and diabetes reflects the well-known North–South divide, with Southern regions faring worse for their combination of lower levels of socio-economic prosperity and poorer healthcare provision (Benassi et al., 2025). A completely different picture emerges by mapping mortality rates from cancer, which instead highlights the disadvantage, particularly for men, of the more industrialised and economically developed Northern regions. Divino et al. (2009) illustrated similar patterning with a finer level of detail, breaking down mortality rates at the provincial level by gender, leading cause, and large age-class. Using a Bayesian spatial approach, which borrows information from neighbouring provinces, they were able to produce reliable and accurate estimates of mortality rates which would have otherwise been subject to random fluctuations, implicit when studying small population and relatively rare events.

While studies that break down mortality patterns by cause can provide valuable insights into their underlying drivers, which also tend to be spatially patterned (Caselli et al., 2003), data that enables such analysis at a fine geographical detail are not always publicly available due to sensitivity considerations. Demographic research, however, has long documented the link between cause-specific and age-specific mortality illustrating how certain causes play a more significant role at certain ages (e.g, Horiuchi et al. (2003); Meslé (2004); Vallin and Meslé (2004)). This association between cause-specific and age-specific mortality has also been documented for Italy. The rise in life expectancy and the decline in lifespan inequality observed at the national level since the 1960s (Nigri et al., 2022) illustrate how shifts in specific causes of death have been closely tied to particular age groups. In particular, the reduction in cardiovascular mortality above age 45 played a major role at the end of the twentieth century, while cancer has assumed an increasingly important role in contemporary mortality declines. Initially concentrated between ages 30–35 and 70–75, these improvements have more recently shifted toward older ages, roughly 40–45 to 80–85. When cause-specific mortality data are not accessible at the subnational level, thus, the study of age-specific mortality trends and their geographical patterning can provide equally valuable insights into underlying mechanisms, offering an evidence base for place-based public health policy. The present study adopts this approach, relying on age- and sex- specific mortality data at the subnational level and applying Bayesian methods to produce robust estimates of the geographical variation in mortality trends.

## The Data

We analyse mortality data by single year of age (0 to 95 years) for males and females in Italy for the period 2002 to 2019. Data on overall death and populations counts come from populations registers, and refer to 107 Italian Provinces (NUTS3), a geographical and administrative division of the country, with population sizes ranging from over 4 million to less than 80 thousand residents according to the last census. As it is often the case, administrative boundaries vary over time, and some adjustments were necessary to obtain a consistent classification of counts over time. In particular, the series on death counts was reconstructed by ISTAT (Italian National Statistics Office) for the purpose of this study, so that, for both series, the geography is consistent over time and counts refer to the most recent territorial classification. As data come from Population registers, they can be considered precise and of good quality. However, as they refer to territorial units with varying population sizes, they are subject to some degree of random fluctuations, hence the value of a Bayesian framework.

## A Spatially Extended Bayesian Lee-Carter Model

Estimating mortality rates below national levels presents significant challenges, particularly when estimates are broken down by one or more characteristics (such as time, age, gender or causes of death) and/or refer to a very fine geographical detail. The resulting spatial distribution of mortality risk may be heavily influenced by random variations present in the observed data. This randomness can conceal true patterns and lead to misleading conclusions, especially when events are rare. Two different approaches have been used in the literature to overcome this problem, and thus reduce variability in the estimates: the first involves pooling together information, e.g., by averaging across multiple years and/or reducing the level of detail of the estimates; the second relies on borrowing ‘strength’ from related observations (e.g., data points that are close in time and/or space).

The method we propose in this paper falls within the second group. We present an extended version of the classical Lee-Carter model (Basellini et al., 2023; Lee & Carter, 1992), a functional model traditionally used to estimate and forecast age patterns of mortality, to the study of the geographical and temporal variation of age- and gender-specific mortality patterns in Italy for the period 2002 to 2019.

### Model Formulation

We use a Poisson version of the Lee Carter model (Brouhns et al., 2002), with a spatial extension, similar to what proposed in Goes (2024), but further interacted by age group, to the mortality data introduced in Section 3, independently for males and females. Let $Y_{xts}$ represent the number of deaths for age *x*, at time *t* in province *s* with $Y_{xts} \mid \lambda _{xts}\sim \text {Poisson}(E_{xts}\lambda _{xts})$, where $E_{xts}$ is the population at risk and $\lambda _{xts}$ the death rate. We model the log rates as:

1 $$\begin{aligned} \log \lambda _{xts} = \alpha _x + \beta _x\kappa _t + \omega _{sg_x} + u_{r_s} + z_{xts}, \end{aligned}$$

where $\alpha _x$ is a age profile at age *x*, $\kappa _t$ a time effect and $\beta _x$ an age-specific multiplication factor. Further, $\omega _{sg_x}$ represents an age-specific spatial effect, where $g_x$ indicates the 10 year age group (0–10, 11–20, 21–30, ...) in which age *x* falls into. Further, $u_{r_s}\mid \sigma ^2_r \sim \mathcal {N}(0, \sigma ^2_r)$ denotes a region-specific effect with $r_s$ indicating the one, of the 20 Italian regions, that province *s* belongs to. This random effect is included to account for unobserved regional heterogeneity. In Italy, the health care governance and financing during the period covered by the study are predominantly managed at the regional level. As a consequence, two geographically adjacent provinces belonging to different regions may operate under quite different health systems, characterized by different levels of efficiency and resource allocation (Ricciardi & Tarricone, 2021). Including a regional-level random effect therefore captures systematic variation arising from these contextual differences and reduces the risk of biased estimates due to omitted regional factors.

Adjustment for overdispersion is incorporated directly in the model by the inclusion of independent Gaussian random variables $z_{xts}\mid \sigma ^2_z \sim \mathcal {N}(0, \sigma _z^2)$. As in Wiśniowski et al. (2015) we assume $\alpha _x$ and $\beta _x$ to be mean zero Gaussian random effects with large fixed variance. For the $\kappa _t$’s we chose a random walk of second order whose directed formulation is given as:

$$\begin{aligned} \kappa _t = 2 \kappa _{t-1} - \kappa _{t-2} + \epsilon _{t}, \, t=3, \ldots T. \end{aligned}$$

Here, we assume a uniform prior for $\kappa _1$ and $\kappa _2$. The error terms $\epsilon _t$, $t=1, \ldots , T$, are independently and identically Gaussian distributed with mean zero and standard deviation parameter $\sigma _\kappa$. Notably, a second order random walk is conceptually similar to a first-order random walk with a time-varying drift component.

For each age group $g_x$, the $\omega _{sg_x}$´s follow a BYM2 model

2 $$\begin{aligned} \omega _{sg_x}= \sigma _\omega \left( \sqrt{1 - \phi } \cdot v_{sg_x} + \sqrt{\phi } \cdot u_{sg_x}\right) , \end{aligned}$$

with the aim of stabilizing estimates for provinces with limited data by borrowing information from neighboring provinces while preserving true local differences (Riebler et al., 2016). Here, $v_{sg_x} \sim \mathcal {N}(0, 1)$ denotes an unstructured province effect and $\textbf{u}_{g_x}$ follows a scaled Besag model that allows spatial smoothing between provinces (Besag et al., 1991). In fact, the Besag model assumes that the mean of the province *s* conditional on all other provinces is given by the average over its neighboring provinces, and the variance is scaled by the number of neighbors. The mixing parameter $\phi \in [0, 1]$ measures the proportion of marginal variance $\sigma _\omega ^2$ explained by the spatially structured effect $\boldsymbol{u}_{g_x}$, so that noise can be reduced but systematic differences between provinces are preserved. For details on the BYM2 models, we refer to Riebler et al. (2016). To account for the fact that the graph for Italy is disconnected, we use adjustments as proposed in Freni-Sterrantino et al. (2018).

To ensure identifiability of the model parameters, standard constraints are imposed such that the sum of $\beta _x$ over age is 1, the sum of $\kappa _t$ over time and the sum of $\omega _{sg_x}$ over space is 0 for every age group $g_x$ (Lee & Carter, 1992).

Of note, we assume that the age effects ($\alpha _x$, $\beta _x$) and the time trend ($\kappa _t$) are constant across Italy, while geographic variation is captured through spatial and regional effects. Although it would be possible to allow age and time effects to vary spatially [see, e.g., Belmont et al. (2024)], doing so would substantially increase model complexity and would be difficult to estimate reliably given the available data.

To assess the plausibility of our assumption, we fitted separate classic Lee–Carter models to data aggregated at the level of the five Italian macro-regions (NUTS1), using the demography package in R. The estimated age and time effects (for both sexes), shown in Figure 7 in the Appendix, are broadly similar across macro-regions, supporting the assumption of spatially constant age and time effects.

### Adjustment for Temporal Change in Regional Mortality

In a second model specification we modify model (1) to allow the spatial effect $w_{sg_x}$ to depend on time. We use $w_{sg_xp_t}$ in (2), where $p_t$ indicates one of two time intervals, 2002–2010 or 2011–2019, in which time index *t* falls into. This enables us to address our third research question of whether the geography of mortality remained constant or changed over time. The cutoff point for the time period responds to both substantive and empirical considerations. First, the literature on recent mortality trends in high-income countries suggests a slowdown in mortality improvements following the economic recession (Dowd et al., 2024), although the exact timing varies across countries. We tested different model specifications using 2006, 2008, and 2010 as cut points. While Murphy and Grundy (2022) and McCartney et al. (2022) agree on somewhat earlier signs of such a slowdown for Italy, placing its onset around 2006, our sensitivity analyses indicate that geographical patterning becomes more apparent from 2010 onwards. Second, using 2010 has the advantage of dividing the overall period into two intervals of equal length, with equal numbers of observations, thus avoiding inflation of the variance of the estimates for either period. Ideally, the cutpoint would be estimated within the model, potentially even at province level, for example by incorporating a Hidden Markov model component. However, this would require further methodological development and would substantially increase the complexity of model specification and estimation.

### Bayesian Inference Using inlabru

For Bayesian inference, we assign prior distributions to all model parameters. We adopt recently proposed penalized complexity priors for the variance parameters of all random effects (Simpson et al., 2017), as well as for the mixing parameter $\phi$. Specifically, for all variance parameters we assume the prior condition $P(\sigma < 3) = 0.05$, while for the mixing parameter we set $P(\phi < 0.5) = 2/3$, following Riebler et al. (2016). We also conducted a sensitivity analysis, which indicates that the results are robust with respect to the choice of priors (results not shown).

We use the newly developed R-package inlabru (Lindgren et al., 2024), which extends the popular INLA (Integrated Nested Laplace Approximation) methodology (Rue et al., 2009) to models with multiplicative effects such as those characterizing the Lee-Carter model. The inlabru approach offers a valuable alternative to Markov Chain Monte Carlo (MCMC) techniques by enabling fast and accurate approximate inference. It provides an intuitive framework for specifying complex models, thereby lowering the barrier for applied researchers to adopt Bayesian inference in practice.

## Results

### Mortality Variation by Age and Gender

Through the estimation of the Lee-Cartel model on mortality data for the period 2002–2019, we address the first research question, highlighting the existence of gender- and age-specific trends in the decline of mortality over the period considered.

**Fig. 1:**
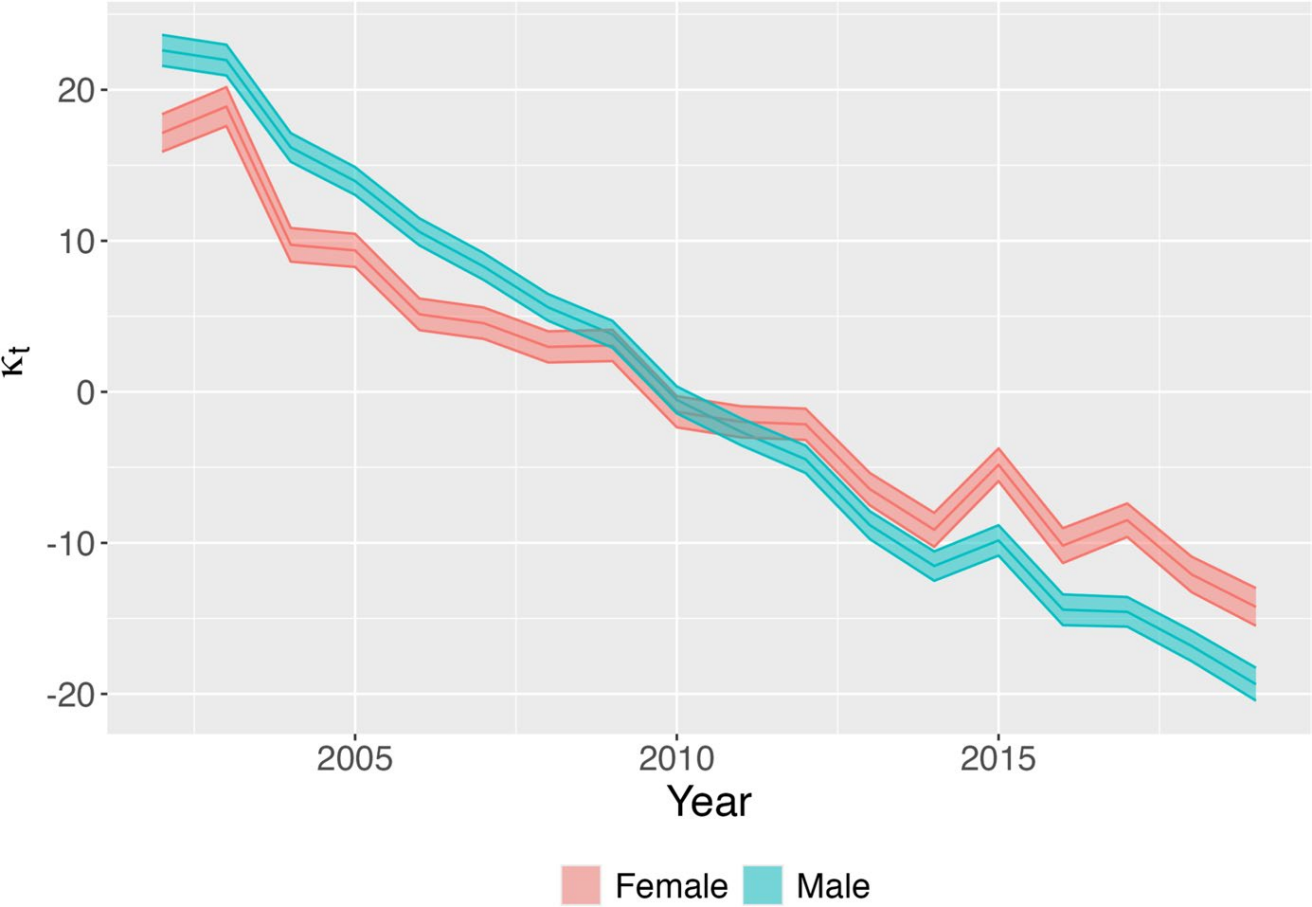
Estimated time trend $\kappa _t$ for males and females. The plot displays the posterior mean (line) together with 95% credible intervals (shaded area) for both males and females

Figure 1 shows the estimated $\kappa _t$, a time index of the general level of mortality. Generally, a linear $\kappa _t$ well captures the historical decline in mortality, and corresponds to a decelerating increase in life expectancy at birth. It also indicates that mortality rates have been decreasing exponentially at their own constant rate over the period of observation. Between 2002 and 2019, the general level of mortality decreased for both genders, although at a faster pace for men–as indicated by the steeper slope of the blue curve. Moreover, the deviation of the estimated $\kappa _t$ from a perfectly linear fit suggests an accentuation of the slowdown in the decline of mortality, particularly after 2014. This accentuated deceleration seems more pronounced for women.

**Fig. 2:**
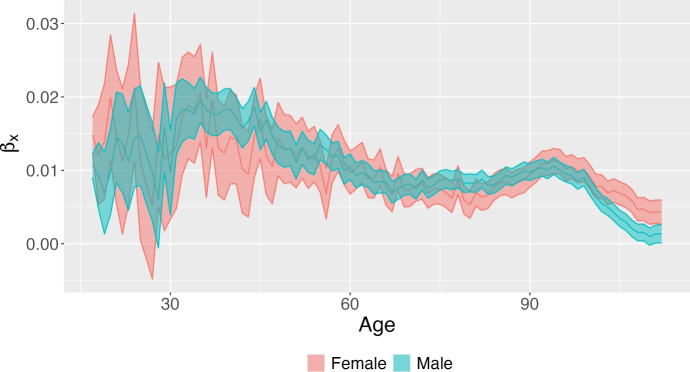
Estimated values for the age-specific multiplicator $\beta _x$: higher values are associated with faster mortality change. The plot displays the posterior mean (line) together with 95% credible intervals (shaded area) for both males and females

Figure 2 displays the estimated $\beta _x$, a parameter that captures the extent of changes in mortality at age *x*, given the overall temporal trend of overall mortality. Higher values of $\beta _x$ indicate a faster change in mortality. Not surprisingly, the decline in mortality rates has been faster at younger ages, although this is also when mortality events tend to be rarer and their estimates subject to greater uncertainty. The pace of mortality decline slows down through adult ages, before rebounding after age 75 (and up to age 95). The age pattern of changes in mortality is quite similar between the two genders, but there are some differences worth noting. The decline has been faster for men than for women at young adult ages (35 to 45) and between 75 and 85, i.e., at ages in which mortality levels for men are higher than for women. Conversely, after age 90, improvements have been faster for women.

**Fig. 3:**
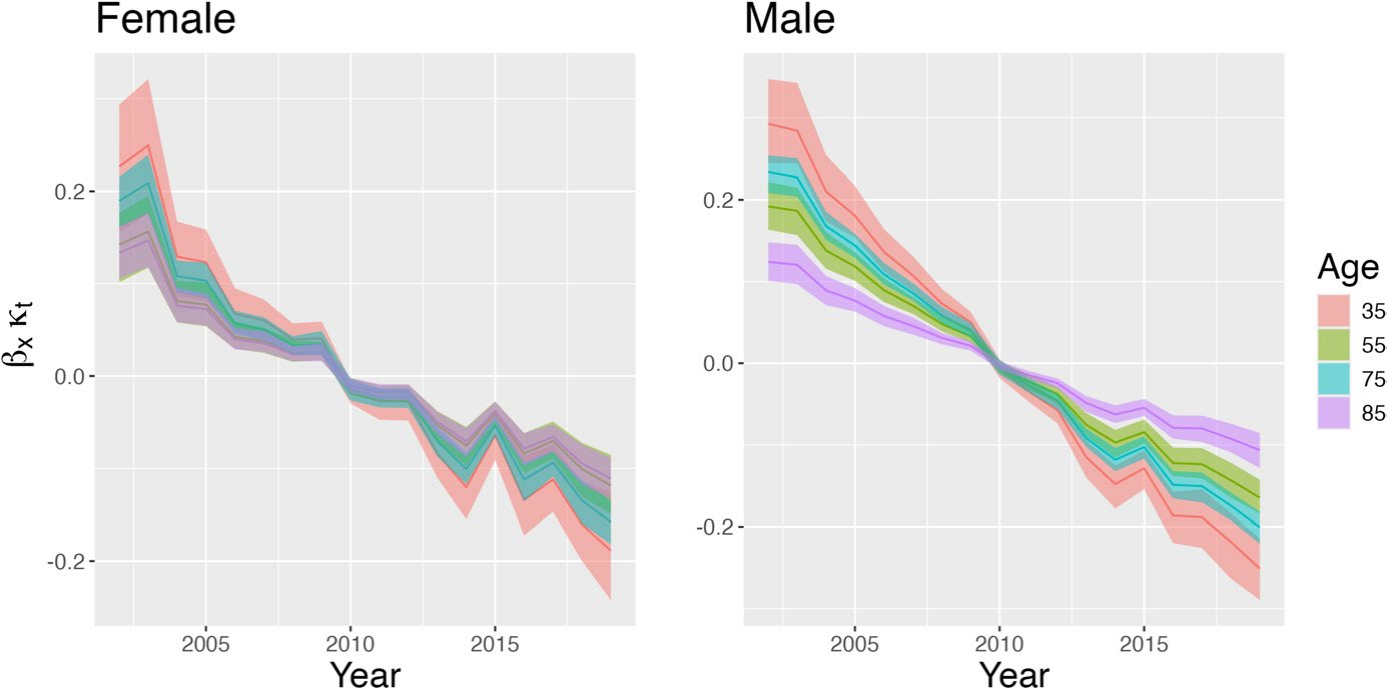
Estimated compound age-specific time effect; $\beta _x\kappa _t$. The plot displays the posterior mean (line) together with 95% credible intervals (shaded area) for both males and females and for selected ages

Figure 3 facilitates the visualization of gender differences in age-specific temporal trends in mortality rates by displaying the compound age-specific time effect ($\beta _x\kappa _t$) for selected ages, and separately for women (left panel) and men (right panel). In line with previous findings, uncertainty is larger for estimates of age-specific effect at younger ages, and particularly so among women, due to the lower frequency of death events for these populations sub-groups.

### Geographical Variation in Mortality by Age and Gender

A key contribution of this study is that it extends the Lee-Carter model through the inclusion of a spatial effect that accounts for geographical variation in the estimates.

**Fig. 4:**
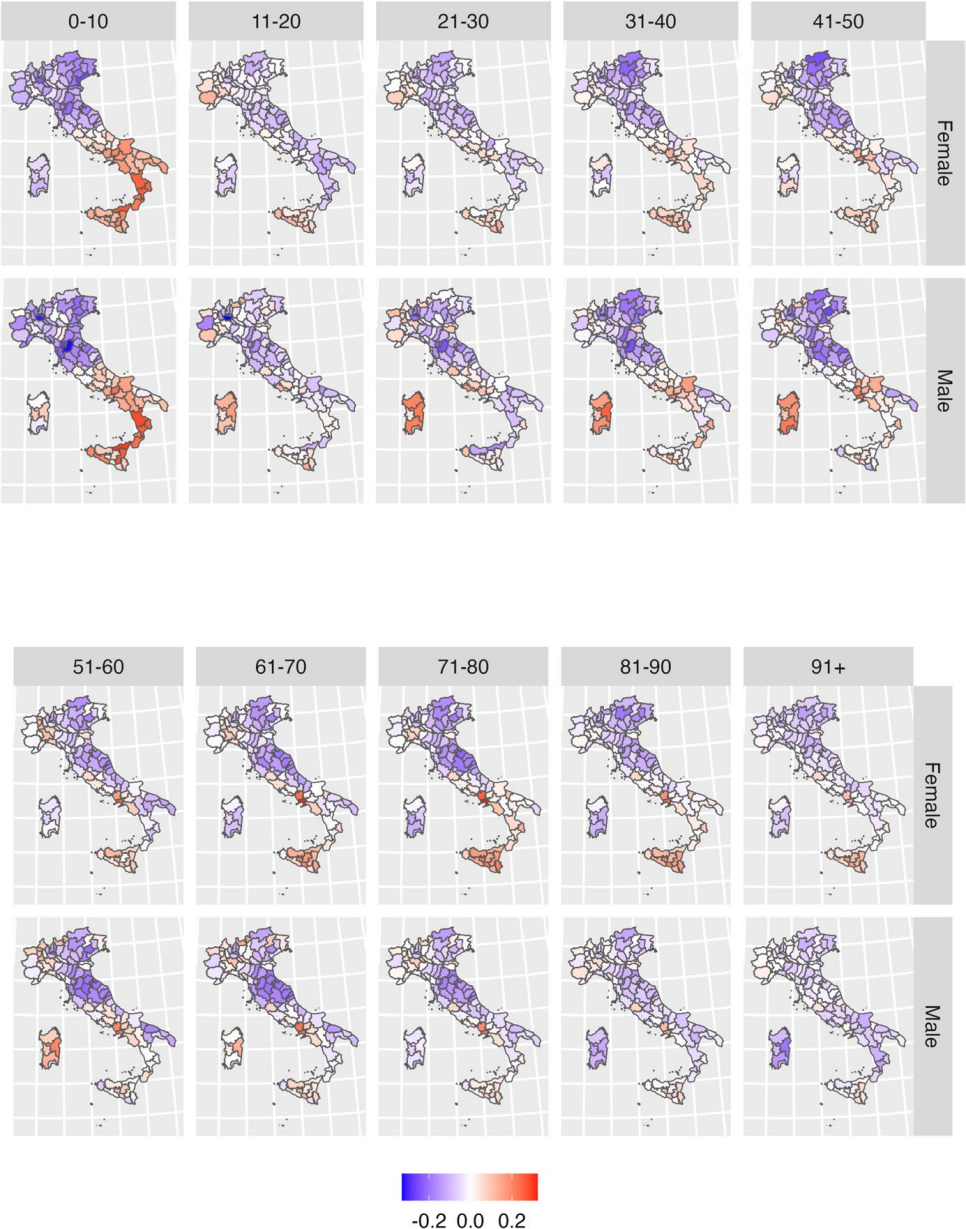
Estimated posterior mean of the spatial effect $\omega _{sg_x}$ for each specific age class and gender. Shades of red indicate provinces with higher-than-average mortality while shades of blue represent provinces with lower-than-average mortality

Figure 4 maps the estimated spatial effect (provincial plus regional) at the provincial level for each ten-year age class, and separately by gender. Shades of red indicate provinces with higher-than-average mortality (for each specific age class and gender); while shades of blue represent provinces with lower-than-average mortality. Figure 8 in the Supplementary Material complements the information by providing a measure of the variance of provincial estimates. In general, it is fair to say that the darker the shades of red or blue, the more likely a province is to exhibit mortality rates which differ from the average.

At first glance, the maps highlight the existence of a gendered spatial patterning of mortality by age, characterized by two key features: first, a greater variability in male mortality rates at younger adult ages; and second a greater variability in femal mortality rates at older ages. The only exception to such gendered geography is observed between age zero to ten, with a marked North–South divide common to both genders, reflecting the same old tale of two Italies with very different levels of wealth, education and provision of health services (Simeoni et al., 2019, 2024).

Among men, geographical differences in mortality levels are particularly pronounced between age 30 to 60, with a mortality disadvantage evident in the provinces of Sardinia and Campania and extending to other neighbouring provinces of the Centre-South (in the regions of Lazio, Calabria, Puglia and Molise). Higher than average mortality is also found in the North-West, while provinces of the Centre-North and North-East represent areas of relatively lower mortality. Geographical disparities become less stark at older ages, although the disadvantage of Naples and other provinces of Campania, as well as that of the North-West, persists. The disadvantage of other provinces in the South fades away; Sardinia, in particular, joins the provinces of Centre-North as an area of comparatively lower mortality for men at age seventy and older.

The geography of female mortality generally confirms the disadvantage of some provinces of the Centre-South and of the North-West, and the relative advantage of the Centre-North and North-East. Geographical disparities in female mortality rates are not as evident at younger adult ages but, contrary to what observed for men, they become more pronounced after age 60. The maps reveal the presence of a hotspot of higher than average mortality in the provinces of Naples and Caserta, and of a general disadvantage extended to all Centre-South provinces (with the exclusion of Sardinia) at older adults ages.

### Widening Geographical Inequalities Over Time

**Fig. 5:**
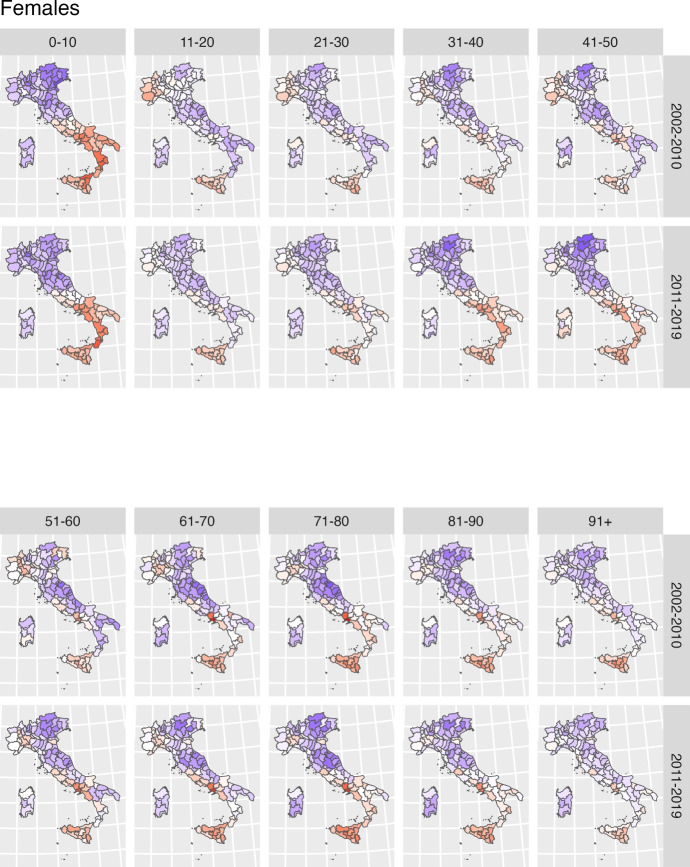
Posterior means for the spatial effect for the second model specification for females. In each row, the top panel represents the first period (2002–2010) and the bottom panel the second period (2011–2019)

**Fig. 6:**
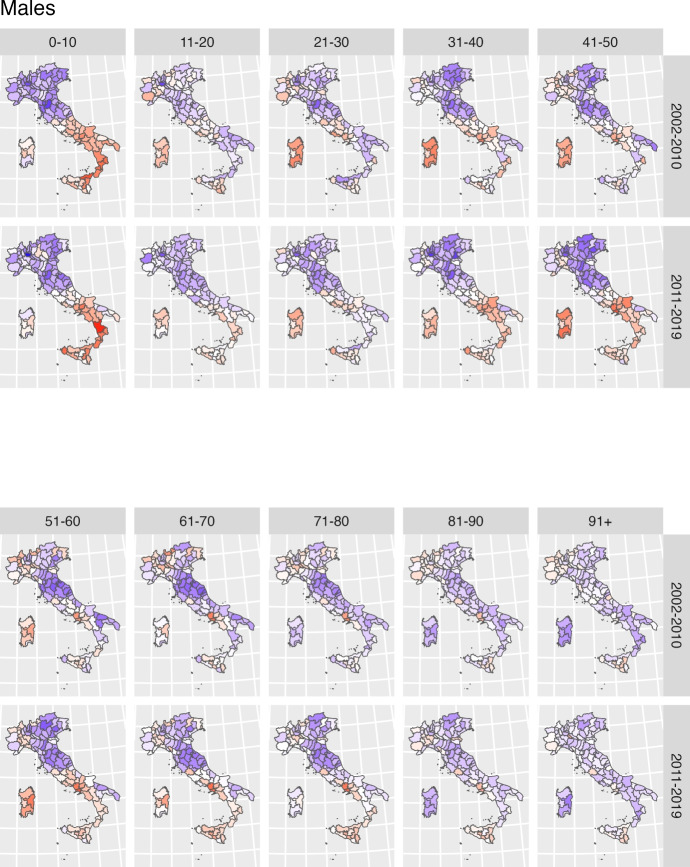
Posterior means for the spatial effect for the second model specification for males. In each row, the top panel represents the first period (2002–2010) and the bottom panel the second period (2011–2019)

**Fig. 7:**
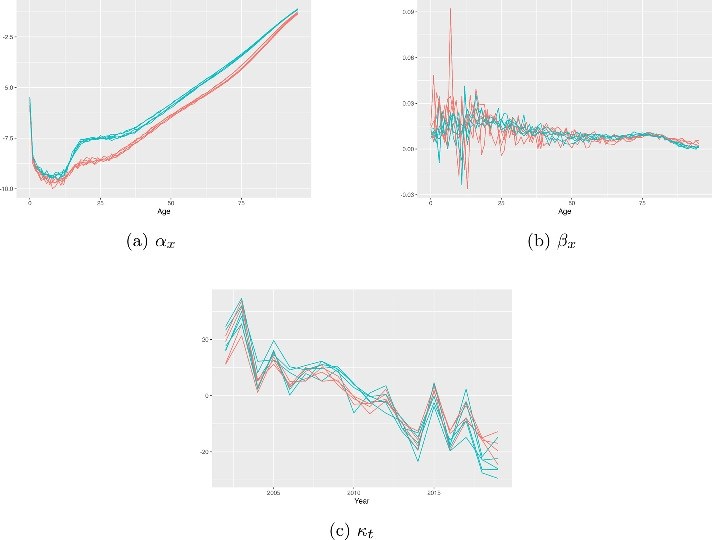
Maximum likelihood estimates of the parameters of a classical Lee–Carter model fitted separately to each of the five Italian NUTS1 macro-regions. Estimates are shown in blue for men and in red for women

**Fig. 9:**
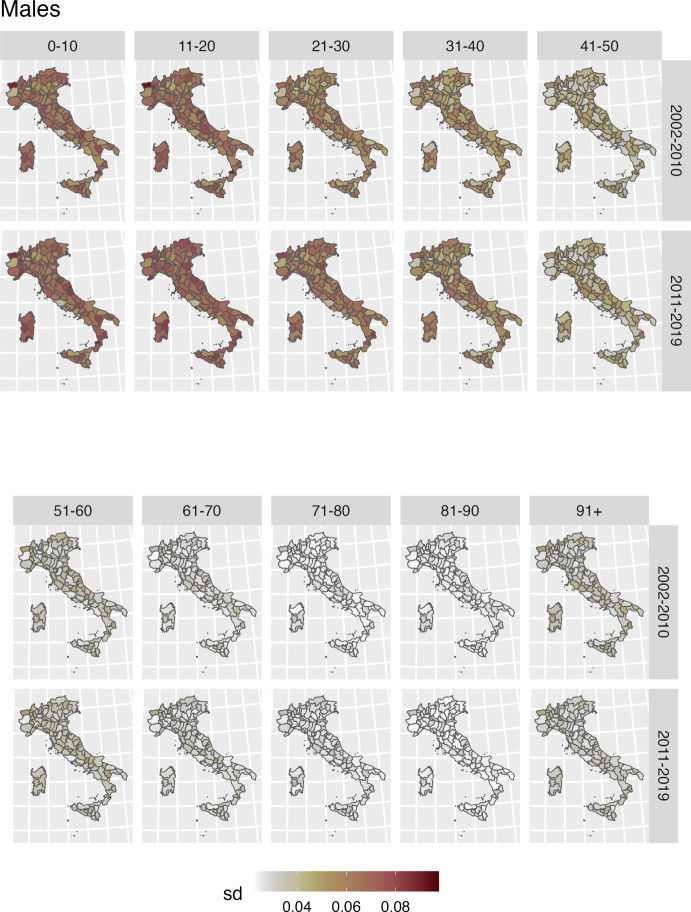
Posterior standard deviations for the spatial effect for the second model specification for males. The top panel represents the first period (2002–2010) and the bottom panel the second period (2011–2019)

**Fig. 10:**
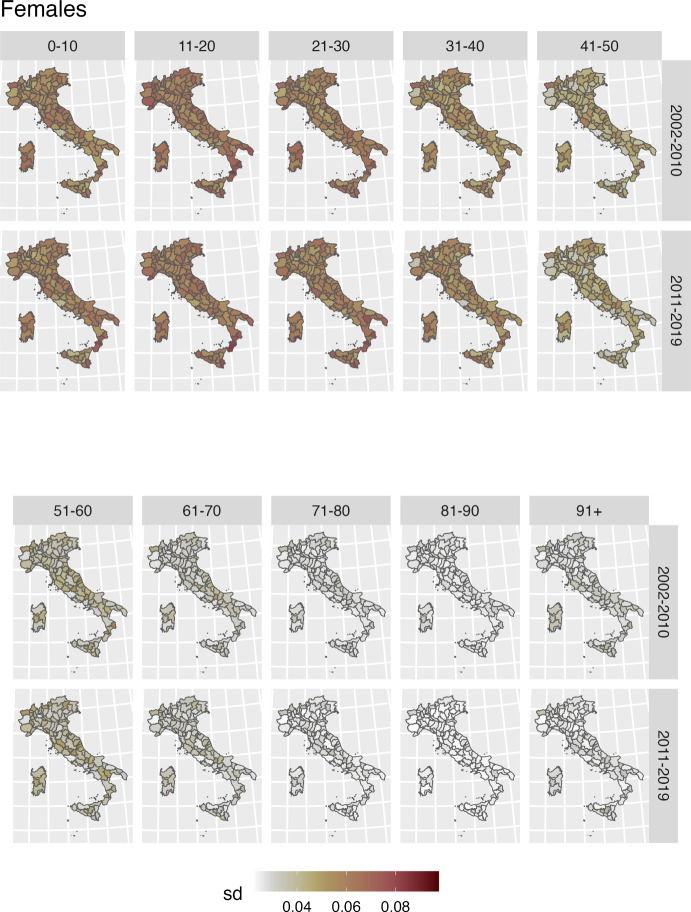
Posterior standard deviations for the spatial effect for the second model specification for females. The top panel represents the first period (2002–2010) and the bottom panel the second period (2011-2019)

The second specification of the Model allows the spatial effect to vary across the two decades, to explicitly address the question of whether the geography of mortality by age and gender has remained constant or changed over time. Figure 5 displays the series of age-specific spatial effects for men and Figure 6 women; for both gender, the top panel represents the first period (2002–2010) and the bottom panel the second period (2011–2019). As before, provinces in blue are to be interpreted as areas of lower than average mortality, and provinces in red as areas of higher than average mortality - with darker shades signifying larger differences. Maps showing the variance of the spatial effect estimates are reported in the Supplementary Material (Figures 9 and 10 ).

A visual comparison of age-specific maps for the two periods reveals both stability and change, which generally hold for both genders: geographical variability in mortality has remained the same (or even slightly reduced) at young (up to age 30) and very old ages (after age 90), but the geography of mortality at adult and older adult ages in the most recent decade has become more heterogeneous.

While there are no signs of reduction of the marked North/South divide observed in mortality rates for children, inequalities in mortality in the teenage and young adult years have narrowed to some extent, mainly because of the disappearance of the relative disadvantage of provinces in North-West. More worrying, however, is the disappearance of the relative advantage of some Southern provinces (in Puglia and Calabria), which in the second decades have mortality levels closer to (or even slightly higher than) the average. Similar patterns are observed for both genders.

Of greater importance, however, is the finding of widening geographical differences in mortality rates at adult ages, with similar patterns again observed for both genders. Both male and female mortality rates show much greater variation in the second decade between age thirty and fifty, with higher-than-average mortality in a much more widespread area of the Centre-South compared to the previous decade, and a more favourable picture in the remaining Centre-North. The only improvement relates to the contraction of the higher-than-average mortality area in the North-West at the same ages.

After age fifty, mortality patterns in the second decade are also shaped by the disadvantage of the Centre-South. For women, this represents the continuation of a long-standing territorial gap, especially evident in Naples, Caserta and surrounding provinces. For men, however, it signals a worsening of survival conditions in the second decade, as excess mortality in the Centre-South becomes more pronounced relative to the rest of the country.

At older ages (80 and above), geographical differences are less evident, and the geography remains largely unchanged between the two decades.

Overall these findings suggest that mid-adult ages constitute the key turning point in recent mortality dynamics, with increasing geographical variability and a marked strengthening of the Centre-South disadvantage.

## Discussion

This study contributes to understandings of geographical inequalities in contemporary mortality trends. It does so by leveraging recent developments in Bayesian spatial and computational statistics to move beyond aggregate data and examine trends in age- and gender-specific mortality rates at a fine geographical level. The focus is on Italy, a country with one of the highest life expectancies in the developed world, but with pronounced social and economic inequalities. These inequalities manifest geographically, shaping health and mortality patterns across a landscape far more complex than the familiar divide between the less economically developed South and the wealthier, more industrialized, and urbanized North (Caselli et al., 2021). As such, the country provides a salient case study to showcase the analytical potential of such methodological innovations for detailed spatio-temporal analysis.

### Recent Trends in Mortality by Age and Gender

More precisely, by applying a spatial extension of the Lee-Carter model to estimate mortality rates across 107 Italian provinces, the study identifies, first of all, clear gender- and age-specific trends in mortality decline over the period 2002–2019. While the general level of mortality decreases for both men and women, the decline seems faster for men. Moreover, and in line with what observed by other studies on Italy (Carboni et al., 2024; Martin et al., 1975) and across other developed countries (Djeundje et al., 2022; Dowd et al., 2024; Ho & Hendi, 2018; Kabir & O’Brien, 2023; Raleigh, 2019), our analyses lend support to the slowdown in mortality decline in more recent times, and particularly so among women. We should always avoid over-interpreting or offering simplistic explanations for what may be merely short-term fluctuations (Luy et al., 2019), but we must not dismiss concerns about the slowdown as simply a sign of a ‘ceiling effect’ among higher performers (Dowd et al., 2024). Rather, if the slowdown reflects genuine shortfalls in achievable mortality improvements, analyses that account for age, gender, context (and cause) specific trends can help identify the underlying drivers and expose inequalities in these processes.

Our estimates, indeed, suggest different paces of decline by age and gender. For example, mortality rates have overall declined faster at younger ages, particularly among men, although it should be noted that this is also the population group for which rates of mortality improvements have been much lower in the second half of the period (Dowd et al., 2024). The pace of the decline then slows down throughout older adult ages up to age 75, and more so for women. This reflects the reduced mortality improvements observed post 2010 after age 55 also by Dowd et al. (2024) and Nigri et al. (2022), which–they argue–are largely related to stalling improvements in mortality from cardiovascular diseases. After age 75, men experience faster decline than women up to age 85, while after age 90 mortality improvements are greater for women.

### The Uneven Geography of Mortality by Age and Gender

While the findings discussed above could have been obtained with a conventional Lee-Carter model, they nevertheless provide useful context and set the stage for the subsequent two questions, which–on the contrary–could have only been addressed through its spatial counterpart, and thus constitute the unique contribution of the study: first, whether these trends manifest evenly across Italy; and second, whether the geography of mortality is becoming more heterogenous at time of slowing mortality progress.

Indeed, the estimation of the Lee-Carter model at provincial level confirms the existence of a complex mosaic, where the red and blue tiles of higher and lower mortality rates do not follow the simple North–South gradient, but appear much more scattered across the country. Moreover, their arrangement does not outline a single geography, but rather varying patterns depending on the combinations of age and gender considered (Barbi et al., 2018; Caselli et al., 2003; Divino et al., 2009; Lagona et al., 2020). For example, while very few provinces in the South (i.e. Naples and Caserta) display consistently higher mortality levels across gender and age groups, the majority changes their relative positioning–such as Sardinian provinces that from areas of higher mortality at younger (male) ages become areas of lower mortality at older (male) ages; or provinces of Puglia and Sicily, which conversely turn into hotspots of higher mortality levels for women at older ages. Even the consistent relative advantage of the provinces of Tuscany, Marche and Emilia Romagna in the Centre North is not as evident at younger and older ages; and that of the Northeast shrinks among men aged fifty and older.

Moreover, geographical variability in mortality is generally greater among younger adult men and among older adult women, suggesting the influence of age- and sex-specific, context-dependent, risk factors. These patterns likely reflect the varying importance of leading causes of death by age and gender, and how economic development, industrialization, and urbanization intersect with disparities in health care services, and differences in lifestyle and behavioural factors, to shape mortality outcomes in Italy (Caselli et al., 2003). Thus, it is not surprising that mortality among women is higher in Southern provinces at relatively older ages, when mortality from cardiovascular diseases is predominant and the role of a sound health system and effective prevention could make the difference (Barbi et al., 2018; Benassi et al., 2025; Caselli et al., 2003; Fantini et al., 2012; Lagona et al., 2020); nor that mortality among men at younger adult ages is higher not just in the more disadvantaged South but also across several Northern provinces, where occupational and environmental risk factors, together with lifestyles such as alcohol consumption and smoking, may be associated with a greater relative importance of cancer mortality (Barbi et al., 2018; Caselli et al., 2003; Federico et al., 2013; Baldi et al., 2026; Nigri et al., 2022).

A further extension of the spatial Lee-Carter model allowed the provincial effect to vary before and after 2010, revealing a widening of geographical inequalities. As a matter of fact, much of the variability discussed in the previous paragraph is driven by patterns observed in the most recent period, whereas the start of the millennium was characterised by a much more homogenous geography. This finding aligns with both earlier studies arguing that a process of convergence in regional and subregional mortality was taking place (Caselli et al., 2003); and with more recent work indicating, on the contrary, the end of such convergence and the widening of spatial inequalities in the second decade of the 2000s, in correspondence with slowing survival improvements (Carboni et al., 2024; Hrzic et al., 2020; Martin et al., 1975; Sauerberg et al., 2024).

In summary, this study extends a commonly applied model to the analysis of temporal trends in mortality rates (Basellini et al., 2023; Lee & Carter, 1992) to account for the existence of a geographical structure to such trends. It overcomes limitations imposed by random fluctuations of death counts across small areas such as the Italian Provinces by adopting a Bayesian approach that borrows strength from neighbouring observations to reduce uncertainty of the estimates; and it demonstrates the value of recent computational advancements by employing inlabru, an R software package that uses Integrated Nested Laplace approximation (Lindgren et al., 2024; Rue et al., 2009) for the estimation of complex spatial models with non-linear effects. Through this approach, it contributes to empirical literature on contemporary mortality trends by highlighting the age patterning of the gendered geography of mortality in Italy, as well as the widening of spatial inequalities in more recent times.

### Limitations and Future Research Avenues

Notwithstanding the important contribution of the present study, there are some limitations that warrant consideration and indicate opportunities for future developments.

First, the analyses presented in this paper focused on the first two decades of the 21st century, highlighting the widening of geographical differences in the more recent decade. While this focus is justified by renewed attention to mortality slowdown and divergence following the 2008 economic recession, a longer-term overview would have enabled to capture the halt to mortality improvement and regional convergence observed since the turn of the 1990s (e.g., Carboni et al. (2024)), a time of significant restructuring of the health system. Unfortunately, at the time of writing, mortality data at provincial level for a consistent geography were only available from 2002; if, and when, longer provincial times series become available, it would be interesting to extend the temporal frame and shed light on subnational variation of mortality at different times of important contextual change. Similarly, while this study only compared geographical variation in mortality before and after 2010, and the exact estimation of a breakpoint in trends fell outwith its scope, future studies could employ different modelling strategies to explicitly estimate breakpoints in subnational mortality trends, thus contributing to the literature on the timing of survival stagnation and reversal (McCartney et al., 2022; Murphy & Grundy, 2022).

Second, while our results suggest that different factors may be conditioning mortality of men and women in the different areas of the country, our analysis did not explicitly assess the association of mortality rates with socio-economic or environmental factors. Existing studies on selected time periods demonstrated, consistently at different geographical scales, the existence of significant associations between mortality and various measures of socio-economic vulnerability, such as for example: unemployment rates at municipal level (Santos Sánchez et al., 2020), a composite index of socio-economic vulnerability at provincial level (Barbi et al., 2018), absolute and relative income at regional level (Dallolio et al., 2012). The spatial Lee-Carter model could indeed have been extended to include geo-referenced covariates. However, identifying relevant and insightful variables collected annually, in a consistent format over an extended period, and at the provincial level, is not straightforward, and would have suffered from comparability issues due to the changing provincial boundaries over time.

More interestingly, operating within a Bayesian framework would have allowed us to produce accurate and reliable estimates at even finer levels of detail as, for example, disaggregating mortality rates also by main (or grouped) causes of death. Unfortunately, the risk of disclosing personal information meant that the Italian National Institute of Statistics could not grant us access to such data. We would welcome future collaborations with National Statistical Institutes on the geographical analysis of age- and cause-specific mortality. Such granular estimates would contribute to uncover the social, structural and environmental nature of place-specific drivers of mortality (Barbi et al., 2018; Caselli et al., 2003; Baldi et al., 2026), providing invaluable information to tailor place-based public health policies and interventions to the populations most at risk.

Lastly, this study estimated the spatial Lee-Carter model separately by gender, as it is common praxis. In principle, it would have been possible to break down the population by other socio-economic characteristics (such as educational level)–thus allowing an exploration of social as well as geographical inequalities. While often information on individual characteristics is not routinely collected or accurately recorded in vital events data, future applications of the method could leverage the opportunity provided by their linkage to census records, or to other administrative database. These data sources offer key advantages for studying health and mortality inequalities, including large sample sizes that enhance precision–especially for rare conditions or minority groups–and long timeframes well suited to tracking changes over time (Keenan et al., 2022). Mortality rates calculated on linked/register data also rely on the same source for status variables in both their numerator and denominator, thus producing more reliable estimates.

Several countries in Europe routinely link their vital events data to census or population register–see, for example, Keenan et al. (2022) for a showcase of studies on spatial and social inequalities in health and mortality using register data from Sweden, Finland, Belgium, Lithuania and from the four constituent countries of the United Kingdom. The Italian National Institute of Statistics does not, to our knowledge, systematically link vital events to Census or Population Registers but has often performed ad-hoc linkages contributing valuable insights to the study of mortality inequalities. For example, the linkage of mortality data for the years 2012–2014 to the 2011 Population Census (ISTAT, 2016; Murtin et al., 2017) showed that low education is, as expected, a key driver of premature mortality but with differences in severity by gender, geography and cause of death. While educational inequalities in alcohol and tobacco related deaths are particularly pronounced among men, diabetes is associated to larger inequalities among women, particularly in the South (ISTAT, 2017). Given the value of these data linkages, along with advances in computational and statistical methods that address challenges in complex spatio-temporal models and sparse data, we strongly support the Institute’s continued efforts^1^ to integrate vital records and population registers for tracking social and spatial inequalities in mortality.

## Conclusion

Our study demonstrated the value of such granular approaches, highlighting the widening of geographical inequalities in mortality rates after 2010, with a distinct age and gender patterning, being more pronounced at working ages for both men and women, suggesting a compound effect of the deterioration of the economic circumstances and reduced healthcare spending. Recent and future trends may be further compounded by the mortality shock and the long-lasting health consequences of the Covid-19 pandemic. The future of mortality in high-income countries is–at best–uncertain (Dowd et al., 2024). To understand current trends and anticipate (and plan for) future ones, it is thus of even greater importance to move beyond aggregate data and analysis to understand how mortality varies between population groups and across regions, and to gain insights on the underlying factors responsible for such variation that may be holding back progress for some.

## Figures and Tables

**Fig. 8:**
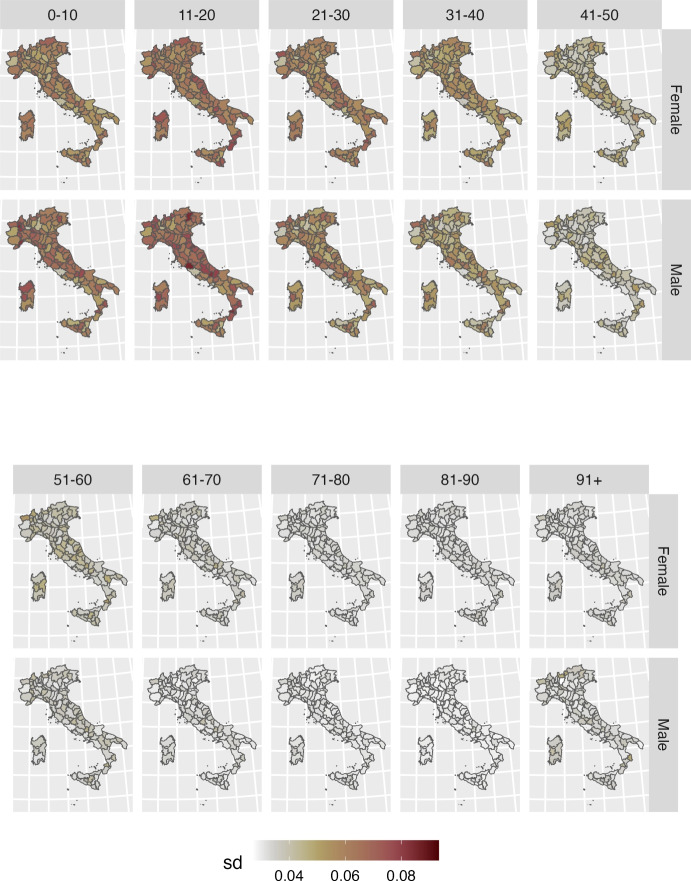
Estimated posterior standard deviation of the spatial effect $\omega _{sg_x} + u_{r_s}$ for each specific age class and gender. Darker colours indicate provinces with higher uncertainty

## Data Availability

We used mortality data by gender, age, year and province for Italy. Data can be requested to the Italian National Statistics Office (https://contact.istat.it/). Simulated data and the code to replicate the analyses can be found at https://github.com/smar-git/Mortality_Italy.

## A Results from a Classic Lee-Carter Model

See Fig. 7

## B Uncertainty maps

See Figures 8, 9, 10

## Footnotes

1 for example, we are aware of the Mortality Inequality Database 2019–2020, linking deaths from the National Register of Causes of Death to the National Base Register (e.g., Feraldi et al. (2025)); and of ongoing work integrating the Istat Population Register with the Income Statistical Register.

## References

[CR1] Abrams, L. R., Myrskylä, M., & Mehta, N. K. (2023). The double jeopardy of midlife and old age mortality trends in the United States. *Proceedings of the National Academy of Sciences,**120*(42), Article e2308360120. 10.1073/pnas.2308360120 PMC10589701 37812715

[CR2] Balia, B., Brau, R., & Marrocu, E. (2018). Interregional patient mobility in a decentralized healthcare system. *Regional Studies,**52*(3), 388–402.

[CR3] Barbi, E., Racioppi, F., & Casacchia, O. (2018). Cause-specific mortality as a sentinel indicator of current socioeconomic conditions in Italy. *Demographic Research,**39*(21), 635–646.

[CR4] Basellini, U., Camarda, C. G., & Booth, H. (2023). Thirty years on: A review of the Lee-Carter method for forecasting mortality. *International Journal of Forecasting,**39*(3), 1033–1049.

[CR5] Belmont, J., Martino, S., Illian, J., & Rue, H. (2024). Spatio-temporal occupancy models with INLA. *Methods in Ecology and Evolution,**15*(11), 2087–2100. 10.1111/2041-210X.14422

[CR6] Benassi, F., Tomassini, C., & Di Felice, G. (2025). Spatial heterogeneities or inequalities? Health care supply and demand of the older population in Italy. *Applied Spatial Analysis and Policy,**18*(1), 44.

[CR7] Besag, J., York, J., & Mollié, A. (1991). Bayesian image restoration, with two applications in spatial statistics. *Annals of the Institute of Statistical Mathematics,**43*, 1–20.

[CR8] Brouhns, N., Denuit, M., & Vermunt, J. K. (2002). A poisson log-bilinear regression approach to the construction of projected lifetables. *Insurance: Mathematics and Economics,**31*(3), 373–393.

[CR9] Capocaccia, R. & Caselli, G. (1990). Popolazione residente per età e sesso nelle province italiane. Anni 1972-1981. Technical Report 2, Rome, pp.251, Fonti e strumenti, Università degli studi di Roma “La Sapienza”, Dipartimento di Scienze Demografiche.

[CR10] Carboni, G., Salinari, G., De Santis, G., & Benassi, F. (2024). Mortality evolution in Italy: The end of regional convergence? *Genus,**80*(1), 28.

[CR11] Caselli, G. (1983). Il contributo dell’analisi per generazioni allo studio della geografia della mortalita. *Genus,**39*(1/4), 37–60. 12266123

[CR12] Caselli, G., Cerbara, L., Heins, F., & Lipsi, R. M. (2003). What impact do contextual variables have on the changing geography of mortality in Italy? *European Journal of Population,**19*, 339–373.

[CR13] Caselli, G., & Egidi, V. (1979). La geographie de la mortalite italienne: Differences territoriales et milieu. *Genus,**35*(1/2), 101–153. 12337499

[CR14] Caselli, G., & Egidi, V. (1981). L’analyse des donnees multidimensionnelles dans l’etude des relations entre mortalite et variables socio-economiques d’environnement et de comportement individuel. *Genus,**37*(3/4), 57–91. 12264944

[CR15] Caselli, G., Egidi, V. & Strozza, S. (2021).* L’Italia longeva. Dinamiche e disuguaglianze della sopravvivenza a cavallo dei due secoli*. Il Mulino.

[CR16] Caselli, G., & Reale, A. (1999). Does cohort analysis contribute to the study of the geography of mortality? *Genus,**55*(1/2), 27–59.

[CR17] Culotta, F. (2021). Life expectancy heterogeneity and pension fairness: An Italian north-south divide. *Risks,**9*(3), 57.

[CR18] Dallolio, L., Di Gregori, V., Lenzi, J., Franchino, G., Calugi, S., Domenighetti, G., & Fantini, M. P. (2012). Socio-economic factors associated with infant mortality in Italy: An ecological study. *International journal for Equity in Health,**11*(1), 45. 22898293 10.1186/1475-9276-11-45 PMC3492165

[CR19] Daniele, V., & Malamina, P. (2011). *Il divario Nord-Sud in Italia 1861–2011*. Rubettino Editore.

[CR20] De Dominicis, L., Arbia, G., & De Groot, H. L. (2013). Concentration of manufacturing and service sector activities in Italy: Accounting for spatial dependence and firm size distribution. *Regional Studies,**47*(3), 405–418.

[CR21] Divino, F., Egidi, V., & Salvatore, M. A. (2009). Geographical mortality patterns in Italy: A Bayesian analysis. *Demographic Research,**20*, 435–466.

[CR22] Djeundje, V. B., Haberman, S., Bajekal, M., & Lu, J. (2022). The slowdown in mortality improvement rates 2011–2017: A multi-country analysis. *European Actuarial Journal,**12*, 839–878.

[CR23] Dowd, J. B., Polizzi, A., & Tilstra, A. M. (2024). Progress stalled? The uncertain future of mortality in high-income countries. *Population and Development Review,**51*(1), 257–293.

[CR24] EUROSTAT. 2025. Mortality and life expectancy statistics. Accessed online on 19 Mar2025. https://ec.europa.eu/eurostat/statistics-explained/index.php?title=Mortality_and_life_expectancy_statistics

[CR25] Fantini, M. P., Lenzi, J., Franchino, G., Raineri, C., Burgio, A., Frova, L., Domenighetti, G., Ricciardi, W., & Damiani, G. (2012). Amenable mortality as a performance indicator of Italian health-care services. *BMC Health Services Research,**12*(1), 310. 22963259 10.1186/1472-6963-12-310 PMC3506466

[CR26] Federico, B., Mackenbach, J. P., Eikemo, T. A., Sebastiani, G., Marinacci, C., Costa, G., & Kunst, A. E. (2013). Educational inequalities in mortality in northern, mid and southern Italy and the contribution of smoking. *Journal of Epidemiology & Community Health,**67*(7), 603–609. https://doi.org/10.1136/jech-2012-201716https://jech.bmj.com/content/67/7/603.full.pdf. 23596251 10.1136/jech-2012-201716

[CR27] Feraldi, A., Pappagallo, M., Giudici, C., & Frova, L. (2025). Monitoring all-cause and cause-specific mortality inequalities in Italian regions. In A. Pollice & P. Mariani (Eds.), *Methodological and applied statistics and demography* (3rd edn., pp. 653–658). Springer.

[CR28] Freni-Sterrantino, A., Ventrucci, M., & Rue, H. (2018). A note on intrinsic conditional autoregressive models for disconnected graphs. *Spatial and Spatio-temporal Epidemiology,**26*, 25–34. 30390932 10.1016/j.sste.2018.04.002

[CR29] Goes, J. (2024). Bayesian forecasting of mortality rates for small areas using spatiotemporal models. *Demography,**61*(2), 439–462. 38482996 10.1215/00703370-11212716

[CR30] Ho, J. Y., & Hendi, A. S. (2018). Recent trends in life expectancy across high income countries: Retrospective observational study. *BMJ,**1*, 1–2. 10.1136/bmj.k2562 10.1136/bmj.k2562 PMC6092679 30111634

[CR31] Horiuchi, S., Finch, C. E., Meslé, F., & Vallin, J. (2003). Differential patterns of age-related mortality increase in middle age and old age. *The Journals of Gerontology Series A: Biological Sciences and Medical Sciences,**58*(6), B495–B507. 10.1093/gerona/58.6.b495 12807920

[CR32] Hrzic, R., Vogt, T., Janssen, F., & Brand, H. (2020). Mortality convergence in the enlarged European Union: A systematic literature review. *European Journal of Public Health,**30*(6), 1108–1115. 32206793 10.1093/eurpub/ckaa038 PMC7733049

[CR33] ISTAT. (2016). Diseguaglianze nella speranza di vita per livello d’istruzione. Nota metodologica. Accessed online on 18 Aug 2025. https://www.istat.it/tavole-di-dati/diseguaglianze-nella-speranza-di-vita-per-livello-di-istruzione/

[CR34] ISTAT. (2017). La misurazione delle diseguaglianze nella mortalità per causa secondo il livello di istruzione. Anni 2012-2014. Nota metodologica. Accessed online on 18 Aug 2025. https://www.istat.it/tavole-di-dati/diseguaglianze-nella-mortalita-per-causa-secondo-il-livello-di-istruzione-anni-2012-2014/

[CR35] ISTAT. 2024. Indicatori demografici 2024. Accessed online on 18 Aug 2025. https://demo.istat.it/tavole/?t=indicatori&l=it

[CR36] Kabir, Z., & O’Brien, S. (2023). Stalling life expectancy trends in Europe and decomposition analysis of mortality data. *European Journal of Public Health,**33*(2), 160-919.

[CR37] Keenan, K., Kulu, H., & Cox, F. (2022). Editorial introduction: Social and spatial inequalities in health and mortality: The analysis of longitudinal register data from selected European countries. *Population, Space and Place,**28*(3), Article e2411.

[CR38] Lagona, F., Ranalli, M., & Barbi, E. (2020). A model with space-varying regression coefficients for clustering multivariate spatial count data. *Biometrical Journal,**62*(6), 1508–1524. 32307746 10.1002/bimj.201900229

[CR39] Lanfiuti Baldi, G., Nigri, A., Trias-Llimós, S., & Barbi, E. (2026). The decline of deaths of despairin Italy: Unveiling this phenomenon in a new context. *Population Health Metrics,**24*(1), 2. 10.1186/s12963-025-00430-9 41514439 10.1186/s12963-025-00430-9 PMC12801950

[CR40] Lee, R. D., & Carter, L. R. (1992). Modeling and forecasting US mortality. *Journal of the American statistical association,**87*(419), 659–671.

[CR41] Lindgren, F., Bachl, F., Illian, J., Suen, M.H., Rue, H., Seaton, A.E. (2024). inlabru: software for fitting latent Gaussian models with non-linear predictors. arXiv preprint arXiv:2407.00791 [stat.ME].

[CR42] Luy, M., Di Giulio, P., Di Lego, V., Lazarevič, P., & Sauerberg, M. (2020). Life expectancy: frequently used, but hardly understood. *Gerontology,**66*(1), 95–104. 31390630 10.1159/000500955 PMC7026938

[CR43] Mackenbach, J. P. (2012). The persistence of health inequalities in modern welfare states: The explanation of a paradox. *Social Science & Medicine,**75*(4), 761–769. 22475407 10.1016/j.socscimed.2012.02.031

[CR44] Mackenbach, J. P., Bopp, M., Deboosere, P., Kovacs, K., Leinsalu, M., Martikainen, P., Menvielle, G., Regidor, E., & de Gelder, R. (2017). Determinants of the magnitude of socioeconomic inequalities in mortality: A study of 17 European countries. *Health & Place,**47*, 44–53. 28738213 10.1016/j.healthplace.2017.07.005

[CR45] Martin, J., Camarda, C. G., & Riffe, T. (2025). Spatial trends in mortality convergence: The cases of France, Italy, and Spain, 1975–2019: J. Martin et al. *European Journal of Population,**41*(1), 21. 40804587 10.1007/s10680-025-09745-7 PMC12350882

[CR46] McCartney, G., McMaster, R., Popham, F., Dundas, R., & Walsh, D. (2022). Is austerity a cause of slower improvements in mortality in high-income countries? A panel analysis. *Social Science & Medicine,**313*, Article 115397. 10.1016/j.socscimed.2022.115397 36194952 10.1016/j.socscimed.2022.115397

[CR47] Meslé, F. (2004). Life expectancy: A female advantage under threat? *Population & Societies,**402*, 1–4.

[CR48] Murphy, M. J., & Grundy, E. M. (2022). Slowdown in mortality improvement in the past decade: A US/UK comparison. *The Journals of Gerontology: Series B,**77*(2), S138–S147. 10.1093/geronb/gbab220 PMC9154273 35107166

[CR49] Murtin, F., Mackenbach, J., Jasilionis, D., & Mira d’Ercole, M. (2017). Inequalities in longevity by education in OECD countries: Insights from new OECD estimates. OECD Statistics Working Papers, 2017/02. OECD Publishing

[CR50] Nigri, A., Aburto, J. M., Basellini, U., & Bonetti, M. (2022). Evaluation of age-specific causes of death in the context of the Italian longevity transition. *Scientific Reports,**12*(1), 22624. 36587058 10.1038/s41598-022-26907-3 PMC9805442

[CR51] OECD / The King’s Fund ed. 2020. Is cardiovascular disease slowing improvements in life expectancy?, Paris. OECD and The King’s Fund Workshop Proceedings: OECD Publishing.

[CR52] Raleigh V (2019) Trends in life expectancy in EU and other OECD countries: Why are improvements slowing OECD? Health Working Papers, OECD Publishing.

[CR53] Ricciardi, W., & Tarricone, R. (2021). The evolution of the Italian national health service. *The Lancet,**398*, 2193–2206. 10.1016/S0140-6736(21)01733-5 34695372

[CR54] Riebler, A., Sørbye, S. H., Simpson, D., & Rue, H. (2016). An intuitive Bayesian spatial model for disease mapping that accounts for scaling. *Statistical Methods in Medical Research,**25*(4), 1145–1165. 27566770 10.1177/0962280216660421

[CR55] Rue, H., Martino, S., & Chopin, N. (2009). Approximate Bayesian inference for latent Gaussian models by using integrated nested Laplace approximations. *Journal of the Royal Statistical Society: Series B (Statistical Methodology),**71*(2), 319–392.

[CR56] Salinari, G., Benassi, F., & Carboni, G. (2023). The effect of the Great Recession on Italian life expectancy. *Population Research and Policy Review,**42*(1), 3. 36742059 10.1007/s11113-023-09755-5 PMC9884069

[CR57] Santos Sánchez, V., Ruiu, G., Pozzi, L., Breschi, M., & Gonano, G. (2020). Geographical variation in mortality and unemployment in Italy. *Rivista Italian di Economia, Demografia e Statistics,**74*, 109–120.

[CR58] Sauerberg, M., Bonnet, F., Camarda, C. G., & Grigoriev, P. (2024). Mortality convergence in Europe? Spatial differences in life expectancy gains between 1995 and 2019. *Population and Development Review,**50*(4), 1401–1427.

[CR59] Simeoni, S., Frova, L., & De Curtis, M. (2019). Inequalities in infant mortality in Italy. *Italian Journal of Pediatrics,**45*(1), 11. 30635011 10.1186/s13052-018-0594-6 PMC6330401

[CR60] Simeoni, S., Frova, L., & De Curtis, M. (2024). Infant mortality in Italy: Large geographic and ethnic inequalities. *Italian Journal of Pediatrics,**50*(1), 5. 38233856 10.1186/s13052-023-01571-z PMC10795306

[CR61] Simpson, D., Rue, H., Riebler, A., Martins, T. G., & Sørbye, S. H. (2017). Penalising model component complexity: a principled, practical approach to constructing priors. *Statistical Science,**32*(1), 1–28.

[CR62] Vallin, J., & Meslé, F. (2004). Convergences and divergences in mortality: A new approach of health transition. *Demographic Research S,**2*, 11–44.

[CR63] Wiśniowski, A., Smith, P. W. F., Bijak, J., Raymer, J., & Forster, J. J. (2015). Bayesian population forecasting: Extending the Lee-Carter method. *Demography,**52*(3), 1035–1059. 25962866 10.1007/s13524-015-0389-y

